# Variation in the Zinc Finger of PRDM9 is Associated with the Absence of Recombination along Nondisjoined Chromosomes 21 of Maternal Origin

**DOI:** 10.4172/2472-1115.1000115

**Published:** 2016-11-23

**Authors:** Tiffany Renee Oliver, Candace Middlebrooks, Ariel Harden, Nyeisha Scott, Blair Johnson, Jillian Jones, Christin Walker, Corinthia Wilkerson, Sha-Hanna Saffold, Abisola Akinseye, Tunde Smith, Eleanor Feingold, Stephanie L Sherman

**Affiliations:** 1 Department of Biology, Spelman College, Atlanta, GA, 30314, USA; 2 Laboratory of Translational Genomics, Division of Cancer Epidemiology and Genetics, NCI/NIH, Bethesda, MD, 20892, USA; 3 Department of Biology, Morehouse College, Atlanta, GA, 30314, USA; 4 Department of Human Genetics, Graduate School of Public Health University of Pittsburgh, Pittsburgh, PA, 15261, USA; 5 Department of Biostatistics, Graduate School of Public Health, University of Pittsburgh, Pittsburgh, PA, 15261, USA; 6 Department of Human Genetics, Emory University School of Medicine, Atlanta, GA, 30322, USA

**Keywords:** Nondisjunction, PRDM9, Recombination, Down syndrome, Zinc finger, Aneuploidy

## Abstract

Variation in the zinc finger-binding domain (ZFBD) of the protein PR Domain-Containing Protein 9 (PRDM9) is associated with altered placement of recombination in the human genome. As both the absence and altered placement of recombination are observed among chromosomes 21 that nondisjoin, we genotyped the PRDM9 ZFBD among mothers of children with Trisomy 21 in efforts to determine if variation within this region is associated with the recombination-related risk for chromosome 21 nondisjunction (NDJ). In our approach, PCR was used to amplify the ZFBD of PRDM9 and products were then subjected to bi-directional Sanger sequencing. DNA sequencing reads were aligned and compared to the sequence of the PRDM9 alleles previously identified. Chi-Square analysis was used to compare allele frequencies between cases (N=235, mothers of children with maternally-derived Trisomy 21) and controls (N=48, fathers of children with maternally-derived Trisomy 21). Results of our analysis showed that the frequency of PRDM9 ZF minor alleles is significantly increased among women displaying NDJ of chromosome 21 and no recombination on 21q (p=0.02). Even more, when compared to those for the PRDM9 major A-allele, these minor alleles displayed fewer predicted binding sites on 21q. These findings suggest that allelic variation in the ZF of PRDM9 may play a role in the risk for chromosome 21 NDJ by leading to reduced recombination on 21q.

## Introduction

PR Domain-Containing Protein 9 (PRDM9) is exclusively expressed during early meiosis in both males and females [1]. Deletions of the gene in mice results in the production of gametes blocked at pachytene of meiosis I that display a reduced number of Dmc1 loci, a protein that localizes to the sites of meiotic crossovers [1]. The human consensus PRDM9 allele (the major allele, also known as the A-allele) binds a 13 bp motif enriched at human LD-based hotspots, namely, CNCCNTNNCCNC [2]. Further, allelic variation in the ZF (zinc finger) of PRDM9 is significantly associated with differential hotspot usage in humans [3,4]. Carriers of PRDM9 minor alleles display reduced recombination in LD-based hotspots [3]; interestingly, this is not necessarily indicative of reduced rates of genome-wide recombination, but rather the altered placement of recombination [5]. Thus the placement of recombination and the hotspots they give rise to, vary by PRDM9 ZF allele. Collectively, these findings explain, at least in part, the molecular basis for the distribution of meiotic recombination in mammals in which the binding of PRDM9 to specific sequences in the genome targets the initiation of recombination at specific locations in the genome.

The number and placement of recombinant events differ significantly between chromosomes 21 that segregate normally and those that nondisjoin. [6–8]. Thus we questioned whether variation in the ZF of PRDM9 was also associated with the recombination-related risk for chromosome 21 nondisjunction (NDJ). Results of our analysis showed that the frequency of PRDM9 ZF minor alleles is increased among women displaying NDJ of chromosome 21 and no recombination on 21q (p=0.02). Even more, when compared to those for the PRDM9 major A-allele, these minor alleles displayed fewer predicted binding sites on 21q. These findings suggest that allelic variation in the ZF of PRDM9 may play a role in the risk for chromosome 21 NDJ by leading to reduced recombination on 21q.

## Materials and Methods

### Ethical standards

The work presented in this publication was approved by the Emory University Institutional Review Board. All participants provided written consent, which indicated that the individuals agreed for study personnel to proceed with the interview and consented for biological specimens to be obtained from them and their child. All information obtained during participant interviews and related to sample collection were catalogued electronically and de-identified. All procedures performed in studies involving human participants were in accordance with the ethical standards of the institutional and/or national research committee and with the 1964 Helsinki declaration and its later amendments or comparable ethical standards.

### Study sample

#### Mothers of children with Down syndrome

Parents of infants with full trisomy 21 were recruited through a multisite study of risk factors associated with chromosome mal-segregation [9,10]. Parents and individuals with trisomy 21 donated a biological sample (either blood or buccal) from which DNA was extracted. Information on race was self-reported by the mother through an interview. For this study, only those reporting as Caucasian were included to reduce population stratification.

#### Determining stage and origin of meiotic chromosome mal-segregation

Samples were genotyped at 1536 SNP loci on 21q by the Center for Inherited Disease Research using the Illumina Golden Gate Assay. The most centromeric single nucleotide polymorphism (SNP) was rs2259403 and the most telomeric was rs46909248. In order to assess the quality of our genotyping data, Mendelian inconsistencies and sample mix-ups were identified using RelCheck among the trios. In addition, parental genotyping data were used to identify poorly performing SNPs. SNPs that met the following criteria were excluded from our analyses: minor allele frequency (MAF)<0.01, deviation from Hardy Weinberg Equilibrium (HWE) (p<0.01), heterozygosity>0.60 or >10% missingness. We also excluded SNPs on a family-by-family basis if >50% of the genotype data for a proband had low intensity levels.

The parental origin of the meiotic error was determined by establishing the contribution of parental alleles to the proband with trisomy 21. Only cases of maternal origin were included in our analyses. Once the maternal origin of the meiotic error was established, markers located in the pericentromeric region (13,615,252 bp–16,784,299 bp) of 21q were used to infer the stage of the meiotic error, meiosis I (MI) or meiosis II (MII). If parental heterozygosity was retained in the trisomic offspring, we concluded an MI error. If parental heterozygosity was reduced to homozygosity, we concluded an MII error. In this assay, we cannot distinguish between the types of underlying errors that might lead to these specific errors. For example, an error that is initiated in MI and not resolved properly in MII leads to the contribution of sister chromatids to the gamete and would be inferred as a MII error. Sister chromatids that prematurely separate in MI will sometimes lead to an “MI” error and other times to an “MII” error depending on the segregation of the chromatids at MI. Lastly, when all informative markers in the parent of origin were reduced to homozygosity, the origin of NDJ was inferred to be a post-zygotic, mitotic error and excluded from the study.

#### Identification of the number and location of recombination along nondisjoined chromosomes 21

Recombination breakpoints were defined by switches from nonreduction (N) or reduction (R) or vice versa of maternal heterozygosity to proband homozygosity for each marker along the nondisjoined chromosome 21 (e.g. NNNNNNNNNNRRRRRRRRRR). In this example, the location of neighbouring markers indicating the first change from N to R (highlighted in bold) would indicate the location of our recombination breakpoints. In order to ensure that the switch from nonreduction to reduction or vice versa was not due to a genotyping error, a minimum of either one informative STR or eight consecutive informative SNPs flanking the recombination breakpoint were required (the example for informative SNPs is shown above). An exception to this rule occurred when the most proximal or distal informative markers on 21q indicated the presence of recombinant event. In these instances, a minimum of either one informative STR or four consecutive informative SNPs were required to define the breakpoints of recombination (e.g. NNNNNNNNRRRR-telomere).

#### Euploid samples – controls

Fathers of children with full trisomy 21 due to a maternal error, who also self-report as Caucasian served as controls for this analysis. These samples were used to establish the PRDM9 ZF allele frequency distribution among normally segregating chromosomes 21. Our analyses that focus on the placement of recombination are case-only analyses, as we did not have information on the placement of recombination in these male controls and because male recombination differs significant from that in females in general with respect to number and location of events [11].

#### Sequencing and allele classification for cases

PCR using previously published primers (PN1.2F TGAATCCAGGGAACACAGGC, PN2.4R GCAAGTGTGTGGKGACCACA [3] was used to amplify the ZFBD of PRDM9 (224589817:23507724-23528706 *Homo sapiens* chromosome 5, GRCh37.p5 Primary Assembly). PCR products were run on a 2% agarose gel to: 1) verify successful amplification and 2) to determine zinc finger (ZF) repeat number genotype. Based on the location of the primers, a sample with 13 ZF repeats would be 1305 bp long with the size of the PCR product increasing in 84 bp increments with each additional zinc-finger repeat. Bi-directional Sanger sequencing of these PCR products was conducted in order to obtain the sequence for the entire ZFBD. DNA sequencing reads were aligned and compared to ZFBD allele sequences identified by Berg et al. [3] (Genbank Accession numbers HM210983–HM211006) in order to characterize the distribution of PRDM9 ZFBD alleles amongst our population of cases and controls. Assuming Hardy Weinberg equilibrium, 74% if the population should be homozygous for the A-allele, 24% should be different heterozygous and 2% should be non-carriers of the A-allele. In instances where individuals were heterozygous for the ZFBD allele, the allele type had to be inferred by assuming that individuals were carriers of one A-allele. Based on the variation, the sequence of the other allele was identified. In instances where the sequence did not provide evidence that one A-allele was present, the specific allele types were not identified, but the sample was classified as carrying two non A-alleles.

#### Identifying the location of prdm9 predicted binding sequences on chromosome 21

In order to identify the sequence of DNA bound by PRDM9 minor alleles, the PRDM9 ZF DNA sequence was translated to an amino acid sequence using the ExPASy - Translate tool. The DNA sequence bound by each ZF allele was determined using a DNA-binding site predictor for Cys2His2 Zinc Finger Proteins [12]. Briefly, given C2H2 zinc finger protein, this program predicts a position weight matrix representing its DNA binding specificity and displays it as a sequence logo. The sequence logo represents each column of the alignment by a stack of letters, with the height of each letter proportional to the observed frequency of the corresponding amino acid or nucleotide, and the overall height of each stack proportional to the sequence conservation, measured in bits, at that position. The letters of each stack are ordered from most to least frequent, so that one may read the consensus sequence from the tops of the stacks. In cases positions where the sequence is not conserved as indicated by a bit quantity <1.0 or ~50% the height of each nucleotide within the logo, the nucleotide call was considered unknown and assigned a call of “N”. The physical location of each binding sequence on 21q was identified using the Short Match Feature from the UCSC Genome Browser. Genotypes used to establish the predicted binding sequences for normally segregating chromosomes were mothers of French Canadian descent with at least two children. Data for the PRDM9 ZF allele distribution among this population were taken from Hussein et al. [13].

### Statistical analyses

Cases were stratified by the stage of the NDJ error, meiosis I (MI) or meiosis II (MII), and chi square analysis was used to compare the allele frequencies between controls and several etiologically-defined subgroups of cases. A 95% confidence interval was calculated for the major allele frequency for each outcome group. Logistic regression was used to compare allele frequencies between subgroups while controlling for maternal age.

## Results

The primary goal of the present study was to determine if variation in the ZF of PRDM9 is associated with the NDJ of chromosome 21. The PRDM9 ZF genotype and allele distributions for cases and controls can be found in Table 1. Over 29 PRDM9 ZF alleles have already been identified among the European population with the major allele (the A-allele) having a frequency of approximately 86% [14]. Our controls, also of European descent, displayed a similar major allele frequency, 84%. This value is comparable to the major A-allele frequency calculated using the raw data from Hussein et al. [13] which estimated the major A-allele frequency among Caucasian women of euploid children to be ~84%. Interestingly, we did find evidence for an excess of minor alleles among MI cases with no recombination on 21q (Table 1). The point estimate for the major allele frequency among MI cases with no recombination on 21q was 0.68 or 68%.

In order to determine if variation in the ZF of PRDM9 was associated with the NDJ of chromosome 21, cases and controls were separated into non-carriers (individuals with the AA genotype) and carriers of PRDM9 ZF minor alleles (individuals with AN or NN genotype where “N” represents any of the previously identified ZF minor alleles [3,13]). Chi-square analysis was then used to determine if the distribution of carriers and non-carriers of minor alleles differed between cases difference and controls. The only significant difference detected was between controls and MI cases with no observed recombination on 21q (p=0.03, Table 2). Data from logistic regression models suggested that women from this outcome group (i.e. MI cases with no recombination on 21q) were 2.45 times more likely to be carriers of at least one minor allele than controls (Table 3, p=0.02, 95% CI=1.17, 5.40).

As MI cases with no observed recombinants on 21q displayed an increased frequency of carriers of PRDM9 ZF minor alleles, it was possible that minor alleles among this population contained fewer binding motifs on 21q that are recognized by PRDM9. This could lead to reduced recombination on 21q, a major risk factor for the NDJ of chromosome 21. This led us to examine the frequency of PRDM9 predicted binding sites on 21q for the major A-allele as well as for minor alleles detected among MI 0 cases (MI cases displaying no recombination on 21q) that were carriers of one major allele and one minor allele. We decided to focus on this population as recent findings suggest that an interaction between PRDM9 major and minor alleles affects PRDM9 binding activity and thus hotspot activity [5]. Results from this analysis showed that the L24 and L9 alleles and alleles with the same predicted binding sequence as the L24 and L9 alleles (here designated N2 alleles) were most frequently observed among MI 0 cases heterozygous for the PRDM9 Major A-allele (Table 4). In addition, there appeared to be fewer binding sequences on 21q for the L24 and L9 alleles when compared to major A-allele (Table 4).

## Discussion

We found that the frequency of the PRDM9 ZF major allele, also referred to as the A-allele, was approximately 0.84 among controls. This finding is important for two reasons; first it provides an independent estimate of the PRDM9 major A-allele frequency that is comparable to studies that also examined samples of northern European ancestry [3,13]. Second, it provides confidence that our method of inferring alleles, which differed from that of others [3,13], correctly identified major and minor alleles among our cases and controls.

We did not find evidence for an increased frequency of PRDM9 minor alleles among MI and MII cases exhibiting only one recombinant event on 21q. This however does not mean that minor alleles are not implicated in the altered placement of recombination on 21q. In order to address this question our future aims entail increasing our population size. This will enable us to limit our analysis of MI and MII cases to only include those that display recombination within the most distal 3.2 and proximal 6.5 Mb of 21q, regions where recombination is significantly increased among MI and MII errors respectively. Only 28% of MI cases are estimated to display a single recombinant event on 21q [14] and while a significant subset of these cases will display altered patterns of recombination, the overall proportion of MI singles is low which prevents us from examining the relationship between the altered placement of recombination on 21q, PRDM9 minor alleles and the risk for the NDJ of chromosome 21 at this time. The absence of recombination on 21q however is observed in approximately 45% of all maternal meiotic cases of trisomy 21 [14]. Interestingly not only did we find an increased frequency of PRDM9 minor alleles among this population, the minor alleles detected among MI 0 cases displayed fewer predicted binding sites on 21q. This observation is consistent with our hypothesis that the reduction in recombination on 21q observed among nondisjoined chromosomes 21 is caused by reduced PRDM9 binding. However, as only ~40% of LD-based hotspots display the 13 bp motif predicted to be bound by the PRDM9 consensus allele, the strength of this support depends greatly on the chromosome 21 specific relationship between LD-based hotspot and binding sequence location.

## Figures and Tables

**Table 1:** Distribution of PRDM9 genotypes stratified by meiotic stage and number of recombinants.

Outcome Group	A/A	A/N	N/N	Number of participants	Major A allele frequency point estimate	95% CI: Lower Limit	95% CI: Upper Limit
MI errors							
Zero Recombinants	0.53	0.3	0.17	87	0.68	0.58	0.78
1 Recombinant	0.65	0.33	0.02	66	0.82	0.73	0.91
>1 Recombinant	0.67	0.25	0.08	12	0.79	0.56	1.02
MII errors							
1 Recombinant	0.65	0.3	0.06	54	0.8	0.69	0.9
>1 Recombinant	0.69	0.31	0	16	0.84	0.67	1.02
Controls	0.73	0.23	0.04	48	0.84	0.74	0.95

**Table 2:** Distribution of carriers and non-carriers of PRDM9 minor alleles.

Outcome Group	Non Carriers	Carriers	Total	P value
MI Zero Recombinants	46	41	87	0.04
MI One Recombinant	43	23	66	0.5
MI>1 Recombinant	8	4	12	0.94
MII One Recombinant	35	19	54	0.51
MII>1 Recombinant	11	5	16	1
Controls	35	13	48	reference group

**Table 3:** Comparing the odds of being a carrier of minor alleles between cases and controls.

Group	Odds Ratio	P Value	95% CI
Control	N/A	Reference Group	N/A
MI Zero Recombinants	2.45	0.02	1.17, 5.40
MI One Recombinant	1.51	0.32	0.68, 3.44
MII One Recombinant	1.67	0.22	0.68, 3.67

**Table 4:** PRDM9 ZF alleles detected among MI zero cases heterozygous for the PRDM9 major A allele.

Allele	Controls	Cases	Predicted Binding Sequence	Number of Predicted Binding Sequences on 21q
L20*	1	1	CCGCCNCGNCCNC	*
L24,L9****		8	CCGCCGTGNCCNC	7
N **		6	CCGCCGTNNCCNC	36
O1		2	CCGCCGGGNCCNC	16
O2***		1	CGGTTAGCGTGNC	0
O3		1	CGGCCGTGNCCNC	7
O4		1	CGGCCGGGNCCNC	10
B	5		CCGCCGTNNCCNC	36
C***	1		CCNCGGTTAGCGTGN	0
L33	1		CCGCCGTGNCCNC	6
L34	1		CCGCCGTGNCCNC	6
N2****		6	CCGCCGTGNCCNC	7

The predicted binding sequence for PRDM9 major A-allele is CCGCCGTNNCCNC. The values indicted under the columns labelled cases and controls represent the number of heterozygous carriers of the major A-allele. The minor allele for these samples and its predicted binding-sequence is indicated.

* The L20 PRDM9 ZF allele had >100 predicted binding sequences on 21q.

** Samples heterozygous for the major A-allele that contain a minor allele that has a predicted binding sequence identical to the PRDM9 major-A-Allele. These alleles have not been previously reported by previous studies.

*** The O_2_ and C alleles had no predicted binding sequences on 21q.

**** Samples heterozygous for the major A-allele that contain a minor allele that has a predicted binding sequence identical to the PRDM9 L24 allele.

## References

[1] Hayashi K, Yoshida K, Matsui Y (2005). A histone H3 methyltransferase controls epigenetic events required for meiotic prophase. Nature.

[2] Myers S, Freeman C, Auton A, Donnelly P, McVean G (2008). A common sequence motif associated with recombination hot spots and genome instability in humans. Nat Genet.

[3] Berg IL, Neumann R, Lam KWG, Sarbajna S, Odenthal-Hesse L (2010). PRDM9 variation strongly influences recombination hot-spot activity and meiotic instability in humans. Nat Genet.

[4] Baudat F, Buard J, Grey C, Fledel-Alon A, Ober C (2010). PRDM9 is a major determinant of meiotic recombination hotspots in humans and mice. Science.

[5] Pratto F, Brick K, Khil P, Smagulova F, Petukhova GV (2014). DNA recombination. Recombination initiation maps of individual human genomes. Science.

[6] Sherman SL, Takaesu N, Freeman SB, Grantham M, Phillips C (1991). Trisomy 21: Association between reduced recombination and nondisjunction. Am J Hum Genet.

[7] Antonarakis SE, Chakravarti A, Warren AC, Slaugenhaupt SA, Wong C (1986). Reduced recombination rate on chromosomes 21 that have undergone nondisjunction. Cold Spring Harb Symp Quant Biol.

[8] Warren AC, Chakravarti A, Wong C, Slaugenhaupt SA, Halloran SL (1987). Evidence for reduced recombination on the nondisjoined chromosomes 21 in Down syndrome. Science.

[9] Freeman SB, Allen EG, Oxford-Wright CL, Tinker SW, Druschel C (2007). The National Down Syndrome project: Design and implementation. Public Health Rep.

[10] Allen EG, Freeman SB, Druschel C, Hobbs CA, O’Leary LA (2009). Maternal age and risk for trisomy 21 assessed by the origin of chromosome nondisjunction: A report from the Atlanta and National Down Syndrome Projects. Hum Genet.

[11] Oliver TR, Bhise A, Feingold E, Tinker S, Masse N (2009). Investigation of factors associated with paternal nondisjunction of chromosome 21. Am J Med Genet A.

[12] Persikov AV, Singh M (2014). De novo prediction of DNA-binding specificities for Cys2His2 zinc finger proteins. Nucleic Acids Res.

[13] Hussin J, Sinnett D, Casals F, Idaghdour Y, Bruat V (2013). Rare allelic forms of PRDM9 associated with childhood leukemogenesis. Genome Res.

[14] Oliver TR, Feingold E, Yu K, Cheung V, Tinker S (2008). New insights into human nondisjunction of chromosome 21 in oocytes. PLoS Genet.

